# Minimization of prediction errors during cerebral embryogenesis and the emergence of agency

**DOI:** 10.3389/fnsys.2025.1683448

**Published:** 2025-11-21

**Authors:** James Joseph Wright, Paul David Bourke

**Affiliations:** Centre for Brain Research, School of Medicine, University of Auckland, Auckland, New Zealand

**Keywords:** agency, free energy principle, predictive error minimization, cortical embryogenesis, structural model, synchronous oscillation

## Abstract

A theory of self-organization in the central nervous system is described, proposing that additive and dissipative synaptodendritic summation leads to synchronous oscillation as the equilibrium state, thereby underpinning a primary mechanism of prediction error minimization. As a consequence, synaptic connections become arranged in mirror-symmetric paired patterns, wherein exchanges of synaptic flux within each pattern form coupled spatial eigenmodes. The mirror-reflection axis between each pair functions as a Markov blanket that maintains excitatory–inhibitory equilibrium, while multiway exchanges among mirror pairs converge toward overall error minimization and mutual organization. The primary organization of this type is evident in the spinal cord. During cortical embryogenesis, connections develop in topographies interpretable as mirror reflections with broken symmetry, aligning along the radial and circumferential axes of cortical growth, as described by the Structural Model, and subsequently manifest at the millimetric scale throughout the cortex. The proposed framework integrates a diverse range of experimental data and provides an explanatory basis for how generative models with agency can emerge through both species evolution and individual learning.

## 1 Introduction

This study reviews the authors' earlier work on cortical dynamics and self-organization in cerebral embryogenesis in the context of current controversy about the utility of the free energy principle and the importance of prediction error minimization in brain function.

The free energy principle, with its corollary, prediction error minimization, has become influential throughout the brain sciences over the last two decades as a significant modification of earlier top-down vs. bottom-up concepts of brain organization. It generalizes formal parallels between laws of nature, including least action, thermodynamics, and Bayesian inference (e.g., Friston, 2002, 2005, 2008, 2010a,b; Hohwy, 2016; Ramstead et al., 2022), and depends upon the idea that all systems separated by a boundary from a wider environment evolve until they have minimized their perturbation by that environment. This implies error minimization across the boundary, as, in the limit, equal and opposite signals are continuously exchanged, resulting in maximum mutual information. Such a boundary is termed a Markov blanket by Friston (Kirchhoff et al., 2018; Friston et al., 2017; Tschacher and Haken, 2007; Fields and Levin, 2019; Kiebel and Friston, 2011; Levin, 2019; Hohwy, 2016).

Applying the principle to the central nervous system (CNS), it is supposed that an internal representation of the self, acting within the world, arises in the higher centers of the brain as a generative model with the capacity to bring about novel consequences—that is, with the capacity for agency.

Due to the principle's generality and unity, and its implications for natural general intelligence, its importance for the future development of artificial general intelligence is apparent. However, it also faces strong criticism. An early criticism was that of the dark room (Friston et al., 2012; Clarke, 2013; Sun and Firestone, 2020)—that escape from stimulus alone would minimize error. Recent criticisms (Kwisthout and van Rooij, 2020; Mangalam, 2025) assert that the principle is metaphoric and not testable; that the computation of Bayesian priors and posteriors could not be achieved within hierarchical architectures; that intermediate values of these measures could not be stored; and that assumptions made about the statistical properties of pulses and electrocortical waves are unphysiological.

These criticisms conflate high-level cognitive abstractions with basic physiological mechanisms. They would be alleviated if it were shown that neurons minimize prediction error in interactions at all scales, so they are not bound solely by hierarchical interactions; that the mechanisms are robust with respect to the statistical characteristics of pulse and wave generation; and that such neural mechanisms tend to evolve toward agency. What follows is a theoretical account of neuroanatomical order that meets these requirements and could be further tested in connectomics.

## 2 The neural field

We begin with definitions of minimal network characteristics (Wright and Bourke, 2024a,b) necessary to justify the simulations upon which our arguments rely. The network properties overlap with those of classical artificial networks (Hopfield, 1982; Rumelhart et al., 1986; Xie and Seung, 2003), but are not identical. When stochastic approximations are assumed, it does not imply that Gaussian or linear properties are necessary.

### 2.1 Presynaptic flux and Hebbian plasticity

The *n* unidirectional flows of presynaptic flux in a neural field can be represented as *n* elements of a square matrix Φ(*t*), with elements φ_*ij*_, each representing the presynaptic flux received at the neuron at position *i* from the neuron at position *j*, over all pathways.


$$\begin{array}{l} \begin{array}{c} \text{Φ}\left(t\right)=GQ\left(t\right)\; \end{array} \end{array}$$ (1)

*G*(*t*) is a square matrix operator of presynaptic gains and axo-dendritic conduction times, transforming *Q*(*t*), a vector of action potential pulse rates of all neurons, so that in the steady state


$$\begin{array}{l} \begin{array}{c} \varphi_{ij}\left(t+\frac{\left|i-j\right|}{\nu }\right)=\pm \varepsilon_{ij}g_{ij}\rho_{ij}Q_{j}\left(t\right)\; \end{array} \end{array}$$ (2)

*Q*_*j*_ is the pulse rate of the *j*−*th* neuron, υ is the speed of signal spread, and $\frac{\left|i-j\right|}{\nu }$ is the delay from pulse generation to arrival of peak pulse density at pre-synapses on the *i*−*th* neuron, averaged over all routes. Synaptic gains are on three time-scales, ε_*ij*_, *g*_*ij*_, ρ_*ij*_–the transient synaptic efficacy, the slow dynamic synaptic gain, and the structural synapse gains, respectively, with synaptic flux either excitatory or inhibitory.

It is assumed that gains are competitive on all three time-scales (Miller, 1996; Okamoto and Ichikawa, 2000) and consistent with the unification of fast and slow synaptic learning rules proposed by Izhikevich and Desai (2003), combining short-term plasticity (STP) and short-term depression (STD) with the slower and more permanent Bienenstock-Cooper-Monro (BCM) rule. Thus, with ongoing network activity, the rapid and transient synaptic efficacies in response to afferent pulses lead to increasing dynamic and structural gains as the time average of synaptic flux.

### 2.2 Dendritic summation and pulse generation

Transformations of synaptic flux leading to pulse generation can be represented as


$$\begin{array}{l} \begin{array}{c} V_{i}\left(t\right)=\delta {\left(t-\tau \right)}^{*}\sum \varphi_{ij}\left(t\right) \end{array} \end{array}$$ (3)

δ(*t*−τ) is a dendritic delay function convolving the aggregate presynaptic flux to generate the dendritic potential, *V*_*i*_(*t*), and may describe any form of leaky integration. The large numbers of independent non-linear summations at the dendritic membrane are assumed to be stochastically smoothed to an additive approximation, where *Q*_*i*_(*t*) is the efferent pulse rate of the *i*−*th* neuron,


$$\begin{array}{l} \begin{array}{c} Q_{i}\left(t\right)=f_{\theta }\left(V_{i}\left(t\right)\right)\; \end{array} \end{array}$$ (4)

and *f*_θ_ is any sigmoid-like function operating as a probabilistic threshold for action potential generation.

### 2.3 Anti-Hebbian plasticity and steady state

Anti-Hebbian synaptic plasticity, reflecting multiple homeostatic metabolic pathways, acts to normalize excitatory and inhibitory synaptic gains, leaving the relative strengths of Hebbian influences unchanged (Keck et al., 2017). A steady state is maintained in which excitatory and inhibitory flux remain balanced so that where ∑φ_*e*_ is the total excitatory presynaptic flux and ∑φ_*i*_ is the total inhibitory presynaptic flux, then


$$\begin{array}{l} \begin{array}{c} \sum {\text{Φ}}_{e}\to -\sum \varphi_{i}\to constancy\; \end{array} \end{array}$$ (5)

This steady-state assumption applies to summed afferent pulses reaching individual neurons as well as the population average; however, the constancy may vary with the level of network activation.

### 2.4 Goals of this model

From these assumptions about network properties, we must demonstrate how the external environmental field, *E*(*x, y, z, t*), where *x, y, z, t* are spatial and temporal coordinates, is mapped onto the developed form of the synaptic connections. That is, we must explain how synapses that converge to a static state can represent sequences of spatiotemporal sensory and motor images. The electrodynamics of synchronous oscillation provide a starting point for understanding.

## 3 Synchrony

### 3.1 Mechanism of synchrony

Synchronous oscillatory firing (Singer, 1993) of cortical neurons is prominent in the 40 Hz (gamma) range but is apparent over all frequencies. Possible mechanisms for its generation are multiple, as it may arise from identical inputs to separate neurons or through non-linear phase-locking of neurons at high or pathological levels of excitation. However, it can be shown that synchrony emerges in any network with zero-mean summing junctions interposed between elements, even when the network is driven by temporally and spatially diffuse white noise. Principal component spatial eigenmode analysis of simulations of the neural field reveals that the first principal component of the field exhibits widespread zero-lag synchrony with relatively low spatial damping and is the response to the even components of the inputs driving the field. The second principal component is characterized by low-magnitude, asynchronous, highly spatially damped, and rapidly dissipated activity and is the response to the odd components of the inputs (Chapman et al., 2002). These properties can account for the bulk of findings on synchronous oscillation in the brain (Wright et al., 2000). Closely related simulations that also account for other EEG properties, including the background spectrum and major cerebral rhythms (Wright and Liley, 1995, 1996; Robinson et al., 1997, 2001; Rennie et al., 2000, 2002), incorporate prominent zero-lag synchrony as an inherent property.

These numerical findings can be simply explained. Oscillation arises from the to-and-fro exchange of signals between excitatory neurons and local inhibitory neurons. Synchrony is then generated by summation of synaptic afferents at dendritic membranes, as in Equation 3, with a set level as in Equation 5. Synaptic pulse trains that arrive in opposite phase with respect to the sustained mean level cancel in summation, while those in phase summate positively—thus out-of-phase signals are dissipative, whereas in-phase pulses, when exchanged through the neural population, converge to coordinated synchrony at all frequencies. This applies to the summation of both excitatory and inhibitory synaptic fluxes at the membrane, since the inhibitory flux is effectively of the opposite phase to the excitatory flux. This argument is independent of the details of pulse generation and exchange statistics, as only the interactions of odd and even signal components are relevant.

Although point, limit cycle, or chaotic attractor dynamics may appear transiently in single neurons, this model is not restricted to such dynamics. A stochastic process results in synchrony as a ground state of electrocortical activity, consistent with observations that electrocortical waves exhibit energy equipartition and minimal free energy (Wright, 1989).

### 3.2 Synchrony as prototypical predictive error minimization

Since the exchange of signals between any two neurons tends to converge toward their synchronous firing, this is a prototypical example of mutual predictive error minimization leading to the maximization of mutual information, *I*. Roughly measured in linear approximation,


$$\begin{array}{l} \begin{array}{c} I=-\frac{1}{2}\log_{2}\left(1-r^{2}\right)\; \end{array} \end{array}$$ (6)

where *r* is the correlation coefficient of their synaptic flux exchange.

From this simple form of prediction error minimization between individual cells, the formation of geometrically ordered multiway exchanges between cell populations can be inferred, as follows.

## 4 The generation of mirror pairs

### 4.1 Minimization of free energy and flux equilibrium

Hebbian learning will lead to a fall in the variational free energy of presynaptic flux, *F*, as cross-connections form.


$$\begin{array}{l} \begin{array}{c} F=A-C\to 0\; \end{array} \end{array}$$ (7)

where *A* is the total presynaptic flux autocorrelation, and *C* is the total presynaptic flux cross-correlation.

A further assumption is made here—that although the asymptotic limit cannot be reached in life, it will be sufficiently and continuously approached despite perturbation, to such a degree that connections will emerge observably close to those expected at the limit.

At the limit at which *F* = 0 (or approximately, if the limit is sufficiently closely approached), in the exchange of synaptic fluxes φ_*ij*_ and φ_*ji*_, over all synaptic routes between cells at any positions *i* and *j*, and time-lag τ, the sum of their autocorrelation terms becomes equal to the sum of their cross-correlations:


$$\begin{array}{r} \varphi_{ij}\left(t\right)\varphi_{ij}\left(t-\tau \right)+\varphi_{ji}\left(t\right)\varphi_{ji}\left(t-\tau \right)=\varphi_{ij}\left(t\right)\varphi_{ji}\left(t-\tau \right)\\ +\varphi_{ji}\left(t\right)\varphi_{ij}\left(t-\tau \right) \end{array}$$ (8)

According to the free energy principle, the synaptic fluxes now represent the spatial and temporal associations in the network's inputs, and their probability distributions satisfy Bayes' theorem.

Absolute equilibrium requires that energy approach equipartition: φ_*ij*_(*t*) → φ_*ij*_(*t*−τ) → φ_*ji*_(*t*) → φ_*ji*_(*t*−τ). This condition is met when pairs of excitatory cells, or pairs of inhibitory cells, exchange equal and opposite flux at zero lag (τ = 0), and when pairs of cells, one inhibitory, the other excitatory, exchange flux by firing with lag $\tau =\frac{\left|i-j\right|}{\upsilon }$. This is synchronous oscillation, in which excitatory or inhibitory pairs fire in phase at zero lag, and excitatory and inhibitory pairs fire in antiphase at half the period of oscillation. The ground state of universal synchrony is approached in the face of external perturbations.

Equation 8 admits many solutions in which free energy is zero, but energy is not equipartitioned. These cases represent forces perturbing the system and external inputs operating upon the neural network and are analogous to *pdV* forces in equilibrium thermodynamics, where free energy is equivalent to the Gibbs free energy.

### 4.2 Prediction error minimization and free energy gradient

Free energy gradients must vanish as the free energy is minimized—but since the system is under continuing input perturbations, then, where ΔΦ^+^ is a vector representing flux induced by the externally imposed signals, there must arise an oppositely directed vector ΔΦ^−^. In effect, the neural network predicts and neutralizes its inputs with minimal error, so


$$\begin{array}{l} \begin{array}{c} \text{Δ}{\text{Φ}}^{+}\left(t\right)-\text{Δ}{\text{Φ}}^{-}\left(t\right)\to 0\;;\;\frac{dF}{dt}\to 0,\;and\;\frac{d^{2}F}{dt^{2}}\to -ve\; \end{array} \end{array}$$ (9)

Since all fluctuations are minimized, synaptic exchanges in zero-lag synchrony are maximized to the extent possible in the face of ongoing perturbation.

### 4.3 The emergence of mirror pair systems and excitatory/inhibitory balance

Fields of synchrony form the spatial eigenmodes of the network. Opposite and equal compensating asymmetric exchanges cannot be within the same eigenmode system, as this would result in a frozen steady state in which a representation of input time variations would not be possible (This would be the dark room outcome.). Therefore, the minimization of prediction errors requires that spatial eigenmodes develop in paired systems with mirror reversal, each with time-varying flux exchanges between eigenmodes oppositely directed to those in the mirror partner.

Paired mirror systems must also be able to maintain overall excitatory/inhibitory balance, as required by Equation 5. This can be provided by the collision of traveling waves at the line of interaction of the mirror pair, modulated by the adaptations of anti-Hebbian plasticity, and mediated by cross-couplings of all excitatory/inhibitory combinations, as diagrammed in (Wright and Bourke 2024a,b). The line of collision is acting as a Markov blanket, with prediction errors in exchanges over the blanket minimized until equilibrium is reached. The paired structure conforms to the good regulator theorem (Conant and Ashby, 1970), which states that every good regulator must be a good model of the system it is controlling. Thus, each of the paired mirrors is a good regulator of the other. When the mirror pair arrangement is generalized to the multiway interactions of many systems, each approaching approximate mirror symmetry with its neighbors, complicated patterns of mutual regulation and distributed systems of partial generative models can arise, influencing one another.

**Figure 1:**
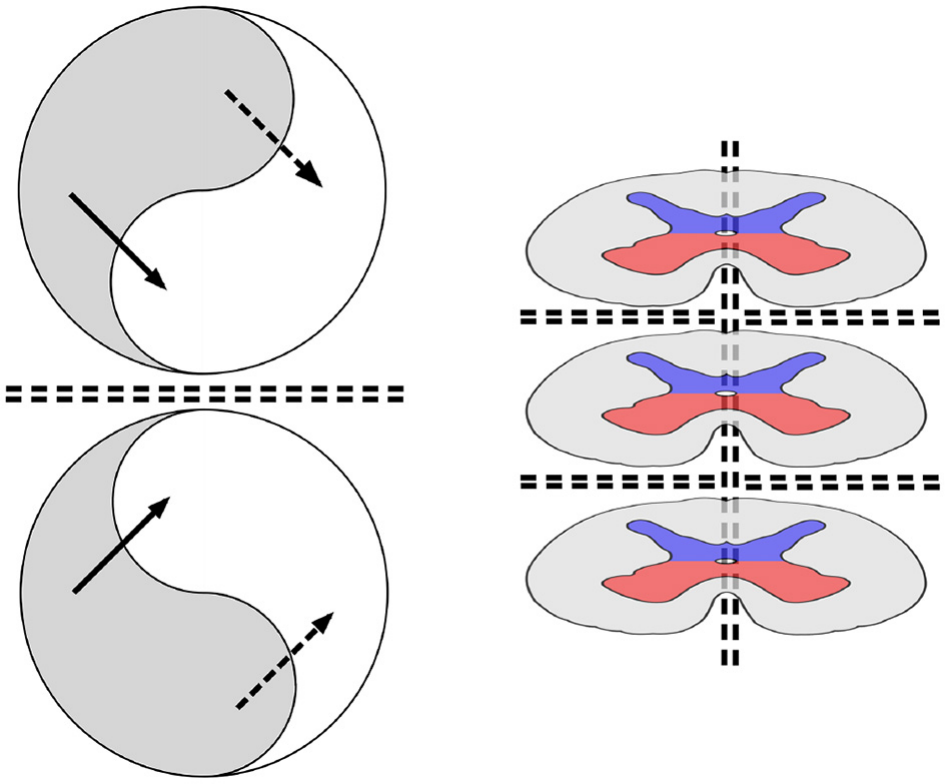
**(Left)** The topology of neural field interactions meeting requirements for minimization of free energy, minimization of prediction errors, and maintenance of excitatory/inhibitory balance. Within each of a pair of mirror-symmetric systems, spatial eigenmodes (represented arbitrarily as yin-yang figures) interact via excitatory and inhibitory cross-couplings (solid and dashed black lines), generating oppositely directed traveling waves that collide at the double-dashed line of symmetry, forming a Markov blanket. **(Right)** The archetypal order of the spinal cord; segmental mirror images; left/right bilaterally symmetric mirror images. Central gray matter is shown in red for somatic and visceral motor outputs and in blue for somatic and visceral sensory inputs. The surrounding system is composed of long neuronal tracts that convey signals over multiple spinal segments to and from the higher centers.

Figure 1 (left) illustrates a system composed of a pair of mirror-symmetric, coupled spatial eigenmodes, each generating oppositely directed, colliding, traveling waves. The diagram shows the topology of the connections and flux exchanges—not a specific topography. The mirror-twin eigenmode systems might be separated by some distance, or their cell soma positions might be interdigitated.

### 4.4 Signal exchanges across Markov blankets

It is emphasized that a Markov blanket is a mechanism for prediction error minimization, but it is not essential that free energy reach the zero limit. Perturbation of the system short of the limit will generate exchanges of signals across the blanket, enabling sequences of activity to be coordinated between mirror pairs, although exchanges across the blanket boundary maintaining excitatory/inhibitory balance result always in convergence toward signal mean and zero-lag synchrony on either side. This requires exchanges that may be mutually excitatory, mutually inhibitory, or involve excitatory/inhibitory cancellation. Exchanges may be out of phase and therefore dissipative, or in phase and conservative. Thus, perturbations of signals across the blankets can be information-rich, and multiway exchanges between assemblies of mirror pairs could organize sensorimotor sequences. This can be observed in the simplest form in the spinal cord.

### 4.5 A prototypical example: the spinal cord

All neural development depends upon cell differentiation in stages, from early precursors into populations of cells with characteristic differences in axonal and dendritic trees and paths of mobilization into anatomical order. Mirror symmetries are apparent from early in the evolution of vertebrates. Figure 1 (right) shows cross-sections of a generic vertebrate spinal cord, introduced here as a harbinger of the development of higher centers.

In Figure 1, right, the dorsal horns of gray matter, colored blue, mark the sites of sensory inputs to each spinal segment. The ventral horns, colored red, are the sites of motor commands sent out via the spinal nerves. The dorsal and ventral horns on each side in a single spinal segment can be regarded as forming a system of coupled spatial eigenmodes. Exchanges across each of the Markov blankets could then maximize the mutual information between sensory and motor signals in the left and right parts of the body, as well as between higher and lower spinal levels. Thus, elementary sensory experiences from within and without the organism, as well as motor expression, would be integrated in a generative model of self-in-the-world, in the simplest sense. Perturbations crossing these boundaries would enable the organization of activity in temporal sequences. This provides a principled basis for the well-studied mechanisms that coordinate locomotion in all creatures with a segmental nervous system, from worms to humans (Danner et al., 2016).

This symmetry could not be generated by the above hypothetical neural mechanisms alone. The structure must be genetically determined and originally selected in evolution by mechanical demands for articulation and the development of segmental reflexes, but it offers a starting point for the evolution of neural mirror symmetries in further development. It is well-established that the organization of the spinal cord extends into the brainstem and subcortex, preserving bilateral symmetry and the extension of structures related to the dorsal and ventral horns of the spinal cord. We assume without proof that the later evolved developments within the subcortical systems could themselves be decomposed into mirror pairs. Instead, we will show how the formation of mirror pairs is apparent in the formation of connections throughout the neocortex.

## 5 Embryogenesis

### 5.1 Selection of neurons and synapses early in the developing brain

Synchronous firing emerges early in neuronal development, coinciding with the establishment of small-world connectivity (Downes et al., 2012; Markov et al., 2011; Vergara et al., 2019; Bassett and Bullmore, 2006). Concurrent apoptosis (Hollville et al., 2019) favors cells that fire with zero-lag synchrony (Heck et al., 2008; Sang et al., 2021), apparently because synchrony promotes high metabolic turnover and resistance to cell suicide (Vergara et al., 2019). Initially, random action potential pulses can generate polysynaptic flows, within which bidirectional monosynaptic couplings may form between cells through symmetric exchanges. Arising first as rostral extensions of the early embryonic spinal cord and brainstem, brain cell precursors that will give rise to the limbic system radiate outward to form the neocortex, in accordance with the Structural Model. As this expansion continues, cortical layers develop in depth, establishing pathways of signal flow and increasingly detailed local patterns of connectivity.

### 5.2 The structural model

The structural model provides an account of the embryogenesis of the neocortex (Barbas, 1986; Barbas and Rempel-Clower, 1997; Barbas, 2015; Garcia-Cabezas et al., 2019, 2020; Sancha-Velasco et al., 2023; Uceda-Heras et al., 2024). Radial lines of cells grow outwards (Sanides, 1962, 1964, 1970) from two primary cell groups related to the hippocampus and olfactory allocortices, respectively, expanding in concentric rings. Cellular and molecular features vary systematically along the radial cortical gradient of laminar complexity (Sancha-Velasco et al., 2023; Garcia-Cabezas et al., 2017; Puelles et al., 2019, 2024; Rakic, 2002, 2007; Barbas and Garcia-Cabezas, 2016; Barbas and Garcia-Carbezas, 2015; Garcia-Cabezas et al., 2019, 2020).

As differentiation proceeds, cell connectivity also undergoes modification under the distance rule (Markov et al., 2011; Vezoli et al., 2021), favoring cross-connections via the shortest and most locally dense pathways (Aparicio-Rodriguez and Garcia-Cabezas, 2023), thereby imposing circumferential connections among the radial lines. The radial lines and circumferential rings become folded into the neocortex, and at the most peripheral limit of development, they include the neurons in the primary sensory and motor cortices.

Along the radial lines of development, centrifugal signals from the limbic system interact with centripetal signals from the special sensory and motor cortex, bypassing each other between cortical layers in a counterflow between a hub-like center vs. the primary cortical areas that establish direct sensory and motor interactions with the environment (Adams et al., 2013). The counterflow is organized according to layers in the cortical depth, with layer complexity increasing from the limbic to the special sensory cortex. The flow outward from the limbic system passes from layers 5 and 6 centrally to layers 5 and 6 and layers 2 and 3 more peripherally. Meanwhile, the counterflow in layer 4, generated by peripheral inputs, interacts with neurons in layers 2 and 3 and is then relayed further via layer 4. Circumferential connections connect layers all-to-all.

The structural model reiterates ancestral neocortical evolution (citations above, Steffen et al., 2022), and the radial hierarchical order can be regarded as an ultimate extension of spinal segmental order. Radial development provides a hierarchical order for minimizing theoretical prediction error (Shipp, 2007; Shipp and Friston, 2022; Bastos et al., 2012, 2020).

### 5.3 Columns and patches at millimetric scale

Further order develops at the millimetric scale in the upper layers, where the superficial patch system (Muir et al., 2011; Muir and Douglas, 2011; Martin et al., 2014), made up of patches of cells that make lateral connections, skipping from patch to neighboring patches in several steps, forms gridworks believed to distribute information between all intracortical locales. Some parts of the cortex are organized into zones (Molnar, 2020; Horton and Adams, 2005), consisting of short-axon neurons surrounded by groups of superficial patch cells, which create variably resolved, often diffuse, macrocolumns. Within these macrocolumns, individual cells are organized according to their stimulus preferences (e.g., Obermayer and Blasdel, 1993). Similar neuron response preferences can be detected whether or not columns are present (Meng et al., 2012), so the presence or absence of columnar structure appears to reflect the degree to which the patch system and intervening shorter axon cells form discrete or merging systems.

Organized response columns in animals, which are clearly defined and have relatively long gestation periods, such as rhesus monkeys, appear before birth (Wiesel and Hubel, 1974). Thus, their formation does not depend upon organized visual inputs. Yet, when deprived of organized visual inputs during the post-natal period, the response architecture collapses (Blakemore and Van Sluyters, 1975; Sherk and Stryker, 1976).

This embryological order can be interpreted as the formation of mirror pairs in a number of distinct topologies, initially driven antenatally by random pulsation and subsequently responding post-natally to ordered inputs.

## 6 All mirrors

### 6.1 Whole brain and interareal approximations to mirror symmetries

In gross anatomy, approximate left/right mirror symmetry extends from the spinal cord to the cerebral hemispheres. Approximate mirror symmetries are also apparent throughout the cerebral white matter, since interareal cortico-cortical connections form U-shaped projections, each area tending to mirror others to which it projects reciprocally.

### 6.2 Hierarchical counterflows as mirror symmetry

The lines of counterflow in the structural model, whereby interactions between signal streams in a hierarchical order occur in cortical layers 3 and 4, can be construed as a mirror reflection of a mirror pair, with the layer 3/4 interaction interpreted as an extended Markov blanket. This aligns with the influential theory of prediction error minimization proposed by Bastos et al. (2012, 2020), in which prediction errors are minimized through excitatory/inhibitory interactions in counterflows.

We suggest that the Bastos et al. models are a special case of a more general and ubiquitous process at all scales—and particularly at millimetric scales—in which paired, mirror-symmetric cells and synaptic arrangements develop, thereby greatly increasing the degrees of freedom for signal interchange and facilitating overall minimization of prediction error.

### 6.3 Simulations of the generation of mirror maps at mesoscale

Detailed accounts of how the selection of developing neurons for maximum synchrony by apoptosis can shape anatomical ordering and the tuning of individual cortical neurons are given in (Wright and Bourke 2013, 2016, 2022, 2023, 2024a,b); Wright et al. (2014).

Simulations (Wright and Bourke, 2013, 2016) were devised to show that continuity between columnar and non-columnar cortex could be explained when total synchrony was maximized and axonal lengths were minimized according to the rules of ultra-small world connectivity. We employed a simple force equilibrium algorithm that leveraged the unit-dimension analogy between mechanical force and synaptic flow, considering the axonal lengths of two populations—specifically, those with long vs. short axons. With an emphasis on small-world cell organization alone, the pattern of cell connections was diffuse. With emphasis on synchrony maximization alone, short axon cells formed clusters surrounded by pools of the long axon cells, the latter in systems with hexagonal, square, or irregular tiling.

With both optimization criteria considered concurrently, simulation outcomes varied depending on the absolute and relative lengths of the short vs. the long axons. Where all axonal lengths were shorter, and when the long axon lengths were closer to those of the short axons, then a more diffuse small-world order predominated. With increasing difference of relative axonal lengths, a more clearly columnar structure emerged. This accounted for the variation in columnar and non-columnar appearances, as well as the ordering of patch cells, with the implication that a single modular system is common throughout, and modular interpenetration in apparently non-columnar areas is made possible by the sparsity of connectivity throughout the cortex.

Using the patterns of cell body positions generated in the simulations, we next reconstructed the patterns of symmetric bidirectional monosynaptic connections that should emerge as synchrony is maximized in the growing network. These matched the distribution of synapses formed by superficial patch cells, with a neighborhood arrangement of cortical columns in mirror symmetry to each neighbor. The closest approximation to mirror symmetry between columns required patch cells in square patterns, with broken symmetry where patch connections were in a hexagonal array.

The requirement to maximize synchrony with bidirectionally symmetric connections in all cases implied a rather strange but necessary arrangement in the connection between patch cells and the cells within each column. The interpenetration of the sparse networks implied that maximization of synchrony among the short axon cells required their linkage in closed loops, while the connections made to the short axon cells by the patch cells would be constrained within arcs radiating from columnar centers. The upshot was that projection from the larger patch cell scale to the shorter axon cell scale would be similar to the projection of a Euclidean plane to a Möbius strip in a Riemann projection. We referred to this as a projection from a “global” to a “local map” scale, which could be represented as follows:


$$\begin{array}{l} \begin{array}{c} P↔p\;where\;p=\left\{\pm \sqrt{-1}k\frac{{\left(P-p_{0}\right)}^{2}}{\left|P-p_{0}\right|}+p_{0}\right\} \end{array} \end{array}$$ (10)

where *P* is a position at the global scale of the patch system expressed as a complex number, and *p* is a corresponding position within one of the local maps. Curly brackets indicate that there is a set of such maps tiling the cortical surface. $\frac{{\left(P-p_{0}\right)}^{2}}{\left|P-p_{0}\right|}$ describes the doubling of the angle in the projection from *P* to *p*, $\sqrt{-1}k$ defines rotation by 90 degrees, and the scale of the projection from global to local scales. Chirality is indicated as ±, and *p*_0_ is the center of a short-axon cell cluster. This implies that a mirroring relationship is created between scales, as well as mirror pairs being generated between adjacent column-like systems throughout the cortex.

### 6.4 Explanatory power of the mesoscale mirror pair model

The above model offers a unitary explanation of the following experimental observations:

- Columnar vs. non-columnar variation (Wright and Bourke, 2016).
- The antenatal development of retinotopic maps and their post-natal loss with deprivation of visual experience (Wright and Bourke, 2013).
- Retinotopic response maps in cortical area V1—Orientation Preference (OP) singularities, linear zones, and saddle points. A Möbius-like internal configuration within a column accounts for the organization of OP from 0 to 180 degrees over the 360 degrees around an OP singularity. Linear zones and saddle points of OP are accounted for by mirror-symmetric reversals with some broken symmetry at columnar boundaries (Wright and Bourke, 2013, 2016).
- Ocular dominance (OD) columns in a square array (Wright and Bourke, 2024a).
- “Like to like” patch cell connections—i.e., the preferential connections of patch cells to columnar cells with a common OP (Wright and Bourke, 2013, 2024b).
- The numerical relationship TFP = object velocity x SFP between preferred spatial frequency (SFP) and preferred temporal frequency (TFP) in individual cortical neurons (Wright and Bourke, 2022).
- The differential distribution of SFP and TFP in relation to OP (Wright and Bourke, 2022).
- OP variation in response to moving line stimuli of given orientation and varied speed, angle of attack, and length (Wright and Bourke, 2013)—as opposed to fixed OP selection on the input pathway alone.
- Recordings made by vertical penetrations of the somatosensory cortex showing that receptor fields for cells exhibit continuities of the fields as the recording electrode is advanced, as well as sudden breaks and reversals of continuity—consistent with the organization of local cell connections in a Möbius-like configuration that spirals within the cortical layers (Wright et al., 2014).

### 6.5 Orderly organization of mirror pairs

As development unfolds, initially highly asymmetric signal exchanges occur between layers 2, 3, and 4 in radial development and between all layers in circumferential development. These exchanges would proceed imperfectly, with later and local self-organization further minimizing total free energy in a manner similar to the formation of eddies in turbulent flow.

**Figure 2:**
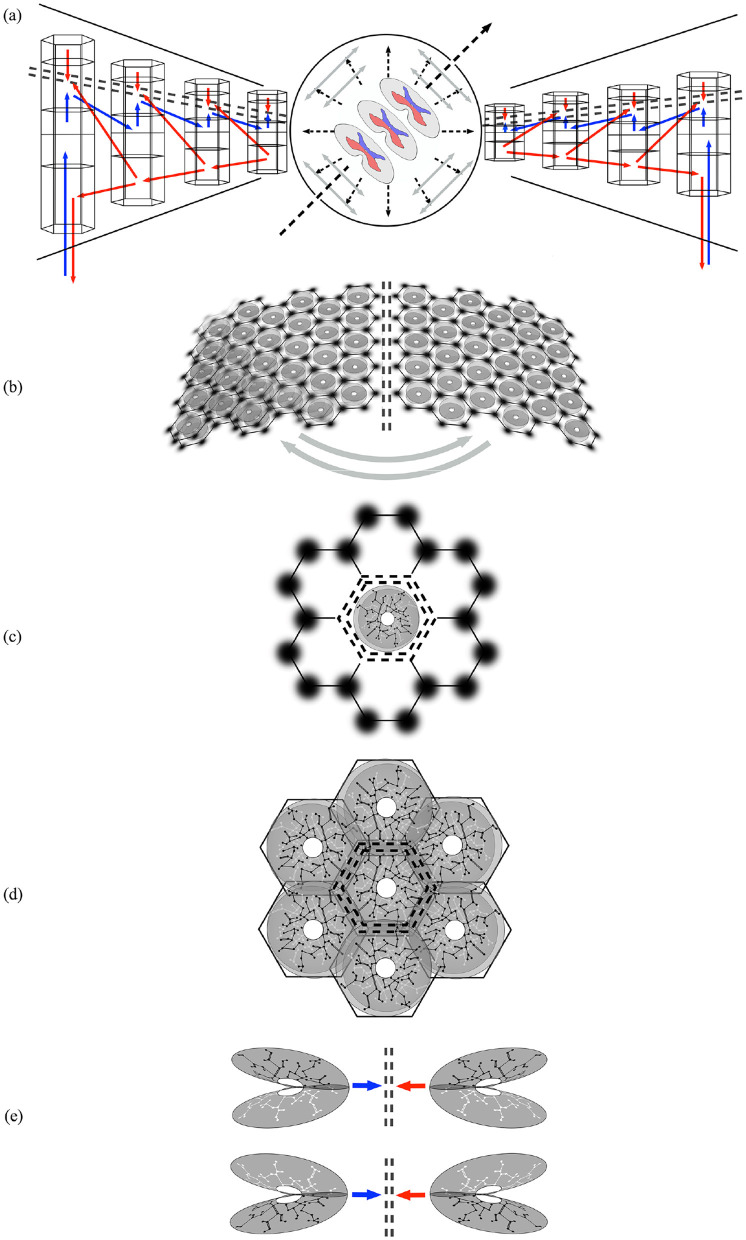
Mirrored representations in approximate developmental sequence. Double black dashed lines indicate lines of mirror symmetry and associated Markov blankets. Red, blue, and gray arrows indicate presynaptic flow along the radial and circumferential lines of development. **(a)** Development along the radial lines of the structural model (see text) indicates the interaction of counterflows at intervening Markov blankets. **(b)** Circumferential development between cortical areas. **(c)** Superficial patch cells, depicted as dark patches, form a communication network that generates local maps in a Möbius strip-like configuration, producing a mirroring between scales at the mesoscale. **(d)** Adjacent local maps, with varying degrees of overlap, interact with each other across homologous mapping positions within Markov blankets. **(e)** Along the lines of radial development, mirror pairs can form in layers 2/3 and layers 5/6.

Figure 2 shows the stages of the proposed development of the mirror pair organization.

In Figure 2a, the central long dashed arrow indicates the orientation of the neuraxis, with the three sections of the spinal cord representing the cord and brain stem. The fine dashed radial lines indicate the direction of embryonic development from limbic origins toward special cortical sensory/motor areas. Lateral intra-cortical and cortico-cortical connections (gray arrows) add circumferential connectivity.

The radial development in depth of the cortical layers of the ventral and dorsal divisions, respectively, is shown on the left and right hand sides from center. Laterally projecting red arrows indicate cortico-cortical connections projecting from higher in the sensory hierarchy to lower, and diagonal blue arrows mark the counterflow of signals from lower to higher in the hierarchy. Small blue vertical arrows mark the onward projections of axons preferentially directed toward the surface from layer 4 to layers 2 and 3, and small red vertical arrows mark counterflows of traffic from layers 2 and 3. Interaction between the counterflows at layers 2, 3, and 4 is characterized as a Markov blanket, indicated by the double-dashed lines.

Figure 2b represents the development of interareal and interhemispheric projection via U-shaped cortico-cortical connections, with approximate mirror symmetry. Superficial patch cells and columnar cells are shown embedded in the cortical sheets (An area on the lower left is shown with overlapping patch connections as a reminder that apparent columnar order can be absent without loss of modular order).

Figure 2c shows the arrangement of superficial patch cells on the cortical surface, forming a communication network between column-like groups of shorter axon excitatory cells that connect in an interpenetrating meshwork, akin to systems of interlocked Möbius strips. Projections from the patch cells to the columns thus form a mirroring between scales at millimetric distances.

Figure 2d illustrates how the maximization of synchrony between adjacent columns necessitates mirrored arrays in neighborhoods, with a degree of broken symmetry imposed by the roughly hexagonal tiling of the patch cell network.

Figure 2e, shown in cortical depth as in Figure 2a, shows how the mirror arrangement would develop as lateral connectivity in layers 2/3 and 5/6, respectively.

## 7 Dimensions and spatiotemporal images

### 7.1 Dimensional consistency in synaptic storage of a generative model

Sensory and motor events can be regarded as images in four-dimensional space-time, arriving in the cortex as image projections to a two-dimensional surface. Specific images in a time-varying three-dimensional world require an object scale/rotation frame of three dimensions plus a position reference frame of three dimensions. The inclusion of information on image velocities then requires a translation within both of these frames. Therefore, when compressed to an effectively flat surface and stored as static cortical synaptic connections, image representations must be defined by sets of twelve points, and these sets must be laid out within an equivalent of a four-dimensional frame—arising as follows.

Where *O* is a pattern of activity at the scale of the patch network, and *o* is the projected image to any given column-like short-axon system, then, according to mapping (10)


$$\begin{array}{l} \begin{array}{c} O\left(P,t\right)↔\left\{o\left(\pm {\left(p-p_{0}\right)}^{2},\;t-\frac{\left|P-p\right|}{\nu }\right)\right\}\; \end{array} \end{array}$$ (11)

Representation of *O*(*P, t*) has been dispersed across a set of synaptic connections that form a four-dimensional reference framework, with positions defined by the complex numbers, *P* (global map) and *p*−*p*_0_ (within each of the set of local maps). Within this frame, associations within sets of twelve neurons can define spatiotemporal images.

Because (10) and (11) are invertible 1:1 maps, and since projections at a large scale are topographically ordered and receive and project to primary sensory and motor areas as topographic 1:1 projections, generative models developed at the mesoscale would maintain topological consistency.

### 7.2 Broken symmetry and sequences of spatiotemporal images

Organizing into mirror pairs would minimize, but not eliminate, broken symmetry. As the gradient of free energy diminishes, in contrast to the idealization of Equation 9, with a degree of asymmetry, an unstable, rather than a stable, fixed point would be approached. i.e.,


$$\begin{array}{l} \begin{array}{c} as\;F\;and\;\frac{dF}{dt}\to 0\;\frac{d^{2}F}{dt^{2}}\to +ve\; \end{array} \end{array}$$ (12)

This may be favorable rather than fatal in functionality. Spatiotemporal images must be stored in sequences much longer than the timescale of intracortical conduction. Longer sequences require cortical areas to activate each other sequentially, creating cognitive order. Therefore, asymmetry would enable the switching and manipulation of spatiotemporal images on longer time scales.

## 8 The evolution of agency

Given the above properties, it is possible to argue that agency can emerge progressively in the evolutionary sequence. The caudal-to-rostral evolutionary sequence can be regarded as a progression from hard-wired to soft-wired connectivity, as is apparent in the progression from lower plasticity to higher plasticity seen in enzyme expression in the radial lines of the structural model (Garcia-Cabezas et al., 2017, 2019; Gomez et al., 2019; Sancha-Velasco et al., 2023). Concurrently, the expansion of mirror pairs in circumferential order provides for parallel and feedback relations with higher degrees of freedom than the hierarchical order alone—a systematic variant of sleep/waking network optimization (Hinton et al., 1995, 2006), in which network connectivity evolves toward optimum in the partially randomized dreaming phase.

We can compare the neocortex, which is considered soft-wired, vs. the limbic, more caudal components, and apply the Nyquist and Shannon relations to measure the channel capacities of both parts of the CNS. The separation into neocortical and limbic systems, as a division between soft- and hard-wired systems, is a convenience, but the argument is general and applicable at various stages as the number of neurons increases in a caudal-to-rostral sequence throughout evolution.

Treating each of *n* synaptic fluxes as binary valued for simplicity, the channel capacity of the entire CNS network, *D*, measured in bits, is as follows:


$$\begin{array}{l} \begin{array}{c} D=n\log_{2}\left(1+\frac{C}{A}\right)\; \end{array} \end{array}$$ (13)

where *C* and *A* are the total cross-correlation and autocorrelations of synaptic flux (Equation 7). $\frac{C}{A}$ is equivalent to a signal/noise ratio, and as the asymptotic limit is approached, *C*→*A* and therefore *D*→*n*.

If the neocortical component is composed of *n*_*neo*_fluxes and a limbic and subcortical component of *n*_*limb*_ fluxes, then there are $2^{n_{neo}}/2$ distinguishable neocortical states in mirror pairs and $2^{n_{limb}}/2$ limbic states.

Survival requires that an organism avoid conditions incompatible with any of the strongly inherited limbic states that constrain and bind its behavior. However, if *n*_*neo*_>*n*_*limb*_, there are $2^{n_{neo}-n_{limb}}$ possible neocortical states available for each limbic state. These neocortical states arise internally through couplings that minimize synaptic free energy. Consequently, as an organism interacts with its environment, various combinations of neocortical and limbic states are generated, and some of the internal neocortical states, by linking to limbic states, gain behavioral expression. The expanded range of states enables the emergence of novel responses that can be tested for their survival potential—either incurring risk or achieving unprecedented success—and, if successful, become incorporated into the organism's behavioral repertoire. This mechanism may underlie the phylogenetic evolution of agency. A parallel process can be proposed for individual development.

## 9 Conclusion

### 9.1 Summing up

We have argued that prediction error minimization is built into the signal exchanges between every neuron in the brain and every other, so that cortical neural networks tend to always maximize synchronous oscillation in the face of ongoing perturbations. Flexible evolution toward this elementary equilibrium at fast, intermediate, and slow time scales of synaptic consolidation would impose the formation of neuronal assemblies in mirror-pair topologies at micro-, meso-, and macroscales, and between scales. This, in turn, allows a four-dimensional generative model to emerge, mapping the four-dimensional interactions of a self in the world.

We have extended the argument to demonstrate that it is consistent with the evolution of the brain being shaped by apoptotic selection interacting with genetic sequences and chemical organizers and, by allowing the addition of extra and flexible information-processing capacity, to progression toward increasing agency.

This theoretical account, if valid, goes some way to countering strong criticisms that have been made of the Bayesian brain and the free energy principle. We have argued that information management is not limited to hierarchical processing, and the primary mechanism of synchrony is robust to the effects of non-linearities and statistical variations that deviate from Gaussian ideals.

### 9.2 Comparisons and further considerations

Our proposal builds upon and extends earlier concepts of brain function and organization.

We have earlier mentioned adherence to the “good regulator theorem,” which requires control systems to model what is controlled and allows for interacting and collaborative control subsystems. The classical Hebbian concept of phase sequences and cell assemblies (Hebb, 1949) could be expressed in terms of coupled spatial eigenmodes. The same notion of self-organization into dynamic groups of neurons with partial computational autonomy, bounded by surrounding Markov blankets, is expressed in the Neuronal Packet Hypothesis (Yufik and Friston, 2016; Yufik, 2019). The present view posits that minimization of prediction error is a universal principle in the interactions of any pair of neurons, as well as in emergent groups with mirror symmetry.

The development of this skeletal concept of neural function into a more comprehensive model of brain function is beyond the scope of this paper; however, areas of interest include, firstly, the interactions between limbic/neocortical systems. Functional correspondences between the structural model and alerting, surprise, habituation, and sleep have been analyzed by Tucker et al. (2022), (Tucker and Luu 2021, 2023), Luu and Tucker (2023), Luu et al. (2024), and Tucker et al. (2025). The activation and regulatory roles of limbic components, positioned as they are at the roots of neocortical development and therefore at the crossroads of signal control, offer a guide to the way the old brain and new brain collaborate in the development toward increasing agency. By minimizing the risk of fatal outcomes, they would influence overall “policy” and, in maneuvering toward optimum adjustment of limbic and neocortical components, would be executing active inference (Ramstead et al., 2021; Kavi et al., 2025). Moreover, the role of sleep proposed in these models draws upon the resemblance to Hinton's “dreaming” networks (earlier cited) and the idea of self-organization through the maximization of synchrony in the presence of noise, with noise and high degrees of freedom assisting movement toward stable basins of attraction. This aligns with the present model's essentially steady-state dynamics, characterized by convergence after perturbation toward maximum synchrony.

Secondly, the interaction of cortical areas, e.g., Yeo networks (Yeo et al., 2011), requires explanation. High asymmetry in the mirror matching of many areas would limit their demarcation via unstable Markov blankets, not only due to the conformation of their boundaries and connections, but also to the mismatching of spatial frequencies resulting from differing fiber ranges for cells in different areas. This would result in relatively weak coupling between some cortical areas vs. others. Such relative isolation between cortical areas might account for cortical area specializations.

Finally, the idea of the brain being organized into systems with mirror symmetries invites comparison with the role of mirror neurons (Bonini et al., 2022). Since mirror neurons show that the neurons of individuals watching other individuals respond as if the observer were experiencing the other's sensations and actions, it might be thought that the term “mirror” may be merely a linguistic link. This is not necessarily the case. The internal generation of mirror neuron activity might itself arise as an imposition upon mirroring of sensory vs. motor activity within the “host.” If so, then there is scope for extending the concept of mirroring more widely than just the individual and the immediate environment, but also to the world of interpersonal and social interactions.

### 9.3 Testing against connectomic data

The occurrence of approximate mirror symmetries at spinal and telencephalic levels is readily apparent, as is the mirroring between cortical areas by cortico-cortical fibers. To a first approximation, the existence of mirror symmetries is uncontroversial—but a critical proposition requiring testing is that superficial patch connections project to shorter axon cells within columnar-sized areas in a manner analogous to the projection of a Euclidean plane onto a Möbius strip. The existence of such structural order is theoretically required for topographic consistency in signal exchanges, and the possible existence of this hypothetical structure is already supported by a range of indirect observations. Three-dimensional schematic diagrams reconstructing the expected connectivity are given in Wright and Bourke (2024b).

It is necessary to show that among the many sparsely connected neurons within a column, patch cells with somas diametrically opposite across any column make synaptic connections in arcs radiating outwards from the central OP singularity and do so over superimposed arcs outwards from the singularity in surface view—but, crucially, that opposite patch cells project to closely interpenetrating, but separate, systems of local cells. This should apply systematically to all patch cell groups around each singularity and should hold for each patch cell's connections to all columns in the vicinity.

Testing hypotheses in this way faces considerable difficulty, particularly with data from small animals or in cortical data obtained from non-columnar cortex. In these cases, the analysis must deal with the interpenetration of the hypothetical columns, as well as the intertwining of local connections within each column. Data for the mouse brain obtained in the MICrONS Project (The MICrONS Consortium, 2025) have a suitable resolution; however, since the mouse is small, the connectivity to be analyzed will face the problem of interpenetration. Mosaic-like connectivity surrounding the line of minicolumns is revealed in the MICrONS mouse data (Miyashita, 2025). This corresponds to the anticipated interweaving of cells found with vertical electrode penetrations (see Section 6.4), but explicit consideration has not been given to patch cells in relation to the interwoven local cells in the connectome data. An easier test case would require data from the columnar visual cortex of a large mammal.

## Data Availability

The original contributions presented in the study are included in the article/supplementary material, further inquiries can be directed to the corresponding author.
